# Shifting Interprofessional Education Pedagogies: Lessons and Implications for Africa

**DOI:** 10.1111/tct.70189

**Published:** 2025-08-25

**Authors:** Farhin Delawala, Yolande Heymans, Christmal Dela Christmals

**Affiliations:** ^1^ Centre for HPE, Faculty of Health Sciences North‐West University, Potchefstroom Campus Potchefstroom South Africa

**Keywords:** development, implementation, individual interviews, IPE programme, qualitative exploratory descriptive

## Abstract

**Introduction:**

Although several theoretical guidelines and evaluations exist for IPE programme development and implementation, practical expert insights remain invaluable. For best practices, guiding future programme design and improving IPE implementation, we aimed to explore the perspectives of 15 international experts on the development and implementation of IPE programmes, including the challenges encountered.

**Methodology:**

A qualitative exploratory descriptive design was used. Fifteen international IPE experts were purposively sampled and individually interviewed using a semistructured interview guide incorporating the key areas of IPE programme development and implementation. Data were analysed using thematic content analysis.

**Results:**

Four themes were identified. Themes include IPE structure in higher education institutions; faculty and student involvement; challenges and opportunities; and evaluation and quality improvements in IPE programmes. International experts prioritised classroom‐based learning, whereas Southern African experts underscored community‐based IPE. IPE‐related challenges revolve around institutional culture, resources, saturated curricula and professional hierarchies, including compulsory IPE inclusion requirements. Yet, these were overcome by opportunities to design innovative IPE programmes while fostering IPE scholarships and research funding.

**Conclusion:**

IPE is gaining international recognition despite common, yet distinct, challenges and opportunities. Classroom‐centric IPE models from HICs are challenged by digital storytelling, home visits and local community collaborations. We found that hierarchies and rigid curricula presented greater challenges than resource constraints. IPE integration in LMICs and underprivileged areas is indeed a transformative strategy for innovation and positive healthcare outcomes.

## 1 Introduction

Interprofessional Education (IPE) is an experience where two or more health professions students learn with, from and about each other and is well established in the medical education community [1]. IPE is known for improving health education, quality healthcare and outcomes. Despite IPE improving Health Professions Education (HPE) and healthcare outcomes, many institutions globally implement isolated, discipline‐specific curricula, limiting students' readiness to address complex healthcare problems collaboratively [2, 3, 4].

IPE is thriving in High‐Income Countries (HIC), while IPE development and implementation in Low‐ and Middle‐Income Countries (LMIC) is limited due to contextual challenges [5, 6]. Hindrances such as nonflexible curricula, professional hierarchy, limited resources, lack of experience and poor institutional buy‐in affect IPE development and implementation [7, 8, 9]. These challenges are global phenomena yet more profoundly experienced in and impacting LMIC [6].

The World Health Organisation (WHO) and other HPE experts strongly advocate for the implementation of IPE programmes [10, 11]. IPE programmes better equip health professions students with competencies thus, enhancing students' readiness to fulfil their corresponding role as members of an interprofessional collaborative care team in the workplace [12]. The WHO's support for IPE is based on reports concerning declining healthcare quality due to poor HPE, including the incapacity of recently graduated medical professionals to work together to address patient problems. Poor healthcare outcomes and a rise in medical errors are significant issues, particularly in LMICs [13, 14].

Theoretical guidelines and evaluations of IPE programmes' development and implementation are well documented by experts [15, 16]. It is expedient to learn about practical experiences from well‐published experts [10, 17]. Conversely, institutions can optimise their existing opportunities by addressing their own challenges and learning from other institutions' challenges. We explored perspectives, including challenges faced, of international experts on the development and implementation of IPE programmes to advise on contextually appropriate approaches for Africa.

## 2 Methodology

### 2.1 Research Design

A qualitative exploratory descriptive design was used to describe experts' experiences in the development and implementation of IPE programmes, as this design is suited for capturing firsthand perspectives of those involved [18].

### 2.2 Sampling

Fifteen experts with recognised contributions to IPE programme development and implementation were purposively selected and invited via email to participate in this study. Consent was obtained by an independent person to avoid coercion, and interviews were scheduled at the experts' convenience. Sampling continued until data saturation was reached.

### 2.3 Data Collection

A semistructured interview guide (Addendum B) and interview checklist (Addendum C) using the 12 key areas of IPE programme development and implementation was developed [15]. Key areas include the following: getting started; adopting a definition, values and principles; formulating outcomes; deciding who will participate and selecting the students and faculty; selecting themes; collaborating in case and activity design and mixing up learning methods; determining levels and stages; facilitating learning; striving to ensure a positive student experience and raising students' expectations; assessing and utilising feedback; evaluating the intervention and sharing the experience. Seven HPE researchers reviewed the interview guide, which was pre‐tested by two educators before conducting interviews. Interviews, lasting between 16 and 60 min, were conducted via Zoom with experts' consent and were recorded to accommodate their diversity and worldwide distribution.

### 2.4 Data Analysis

Data were analysed using thematic content analysis [19] with ATLAS.ti software. Zoom recording's transcripts were verified and corrected by a professional transcriber. ATLAS.ti was used to code the transcripts inductively. The first author conducted the coding, which was then verified through team consensus, ensuring trustworthiness. Similar codes were clustered into subthemes, and subthemes were combined into four main themes, supported by verbatim quotes from experts (Addendum A).

### 2.5 Ethical Considerations

Ethical approval was obtained from the North‐West University's Human Research Ethics committee, and all ethical principles were observed, including informed consent, confidentiality agreements by those involved, voluntary participation without negative consequences, maintaining experts' privacy by allocating code identifiers and consent for audio recordings.

## 3 Results

### 3.1 Participant Information

This study included 15 IPE experts, from Africa (7), Asia (1), Australia (1), Europe (2) and North America (4). Demographic profiles of experts are presented in Table 1.

**TABLE 1:** Demographic profile of participants.

Participants	Gender	Highest qualification obtained	Field of expertise	Country where IPE is practiced
D1	M	Master's degree	Academic innovation; online learning; IPE; IPE publications	United States of America
D2	M	PhD	Faculty development, competency‐based education and sustaining curricular innovations; IPE; IPE publication	South Africa
D3	M	PhD	Curriculum development; co‐ordination; IPE; IPE publications	South Africa
D4	F	Master's degree	Interprofessional collaboration, education and learning	South Africa
D5	F	PhD	Teaching and learning; curriculum development; IPE; IPE publications	South Africa
D6	F	PhD	Teaching and learning; curriculum development; co‐ordination; IPE; IPE publications	South Africa
D7	M	PhD	Curriculum review and implementation; IPE and collaboration: IPE publications	India
D8	F	PhD	Interprofessional working and learning: IPE publications	United Kingdom
D9	F	PhD	Blended learning; collaborative learning; curriculum development; IPE	South Africa
D10	M	Master's Degree	IPE	United States of America
D11	F	Master's Degree	IPE; IPE publications	South Africa
D12	F	PhD	Development and evaluation of interprofessional curriculum; IPE publications	Australia
D13	F	Master's Degree	IPE; blended learning	United Kingdom
D14	F	Master's Degree	IPE; IPE publications	United States of America
D15	F	PhD	IPE and collaboration; IPE publications	United States of America

### 3.2 Themes and Subthemes

Four themes were identified and described: IPE structure in higher education institutions; faculty and student involvement; challenges and opportunities; evaluation and quality improvements in IPE programmes (Table 2). Most themes had two subthemes except for the first theme, which had five subthemes.

**TABLE 2:** Themes and subthemes informing IPE programme development.

Themes		Subthemes
Theme 1	IPE structure in higher education institutions	Definition of IPE
		Motivation for IPE
		Objective of the IPE programme
		Stakeholder involvement
		Components of IPE
Theme 2	Faculty and student involvement	Student involvement
		Facilitator/faculty involvement
Theme 3	Challenges and opportunities	Challenges
		Opportunities
Theme 4	Evaluation and quality improvement in IPE programmes	Suggestions for improvements
		Dissemination of findings
		Evaluation

#### 3.2.1 Theme 1: IPE Structure in Higher Education Institutions

Subthemes described under this theme include how IPE was defined by various institutions, what the motivation was for developing IPE programmes, the objective of the IPE programme, stakeholder involvement and the components of the IPE programmes.

##### 3.2.1.1 Subtheme: Definition of IPE

Seven (D2; D5; D6; D7; D11; D14; D15) of the 15 experts reported using the WHO definition, two (D1; D10) used the Interprofessional Education Collaborative (IPEC) competency domains and three (D3; D8; D12) used the Centre for the Advancement of Interprofessional Education (CAIPE) definition. However, two (D9; D13) generated their own definition while one expert (D4) noted the definition emerged through community engagement.

##### 3.2.1.2 Subtheme: Motivation for IPE

Experts emphasised that patient care and the need for a collaborative approach in Primary Healthcare (PHC) served as motivation for IPE programmes. IPE mitigated conflict, misdiagnosis, medical and communication errors and ethical dilemmas in healthcare service delivery. IPE aimed to facilitate practice‐ready graduates and accreditation requirements in addition to some institutions requiring IPE as part of undergraduate training and curricula.

IPE mitigated conflict, misdiagnosis, medical and communication errors and ethical dilemmas

##### 3.2.1.3 Subtheme: Objectives of the IPE programme

Four experts (D1; D10; D14; D15) were explicit in using IPEC competencies to formulate the aims and objectives of their IPE programmes. All experts stated that the expected outcomes of their IPE programmes included knowledge construction, patient safety, problem‐solving skills, understanding of professional roles, having mutual respect, collaborative practice, ethical practice and teamwork. Emphasis was placed on interprofessional collaboration (IPC) and patient‐centred care, with conflict management skills for successful collaboration.

Emphasis was placed on interprofessional collaboration (IPC) and patient‐centred care with conflict management skills for successful collaboration

##### 3.2.1.4 Subtheme: Stakeholder Involvement

Stakeholders in IPE included institutional members and community partners involved in student engagements. All experts involved individual stakeholders, that is, seconded staff, observers or invitees. Institutional leadership played vital roles in programme development and faculty recruiting. One IPE programme elected ‘IPE champions’ that were selected from different departments to encourage participation, while others gathered inputs from regulatory authorities and stakeholders through seminars and national consultations.

##### 3.2.1.5 Subtheme: IPE Components

Student activities (Table 3) varied across institutions, which included diverse learning. Activities included designating IPE days, international online collaborations, creating care plans, debriefing sessions where students analysed experiences and worked on collaborative skills, standardised case vignettes, roleplay, workshops and simulations. Activities such as world cafes and ‘the amazing race’ aided in building teamwork and problem‐based learning through interactive, scenario‐based exposure. Further learning involved students conducting home visits, community needs analysis, health promotion talks and digital storytelling. Facilitator guides, student workbooks, asynchronous learning materials and multimedia tools formed part of programme delivery. Delivery durations ranged from hours to multiyear implementation depending on IPE structures.

**TABLE 3:** Summary of IPE activities offered and suggested by experts.

Activity fulfilling IPE intention	Intention of learning objective related to IPE Activity				Overarching online and/or in person activity to fulfil IPE intention		
	Knowledge construction	Patient safety	Problem solving	Collaborative practice	World Café	Amazing Race	Community involvement
Case studies	x	—	x	x	x	—	—
Simulations	x	x	x	x	x	x	x
IPE care plans	x	x	x	x		x	x
Collaborative skills development	x	x	x	x	x	x	x
Workshops	x	x	x	x	x	x	x
Community needs analysis	x	x	x	x	x	x	x
Home visits	x	x	x	x	x	x	x
Digital story creation	x	—	—	x	x	—	x

#### 3.2.2 Theme 2: Faculty and Student Involvement

This theme and subthemes relating to coordination, student involvement and facilitator/faculty involvement address the exposure, approach and engagement of faculty and students in IPE programmes. Table 3 provides a summary of student involvement and objectives.

##### 3.2.2.1 Subtheme: Coordination

IPE coordination is essential to guaranteeing students acquiring the skills required for team‐based healthcare practice. Experts underlined both interim and long‐term outcomes such as mutual respect, role clarity and shared values in accordance with IPEC competencies, that is, values and ethics, communication, roles and responsibilities and teamwork. Specific goals including team‐based care, IPC, patient advocacy and conflict management skills were deemed necessary for patient‐centred care.

Collaboration within IPE promotes equal involvement, shared domains of practice and the creation of collective patient care plans. IPE piloting was recommended, starting with facilitators, then moving on to students and incorporating student input through tools such as questionnaires. By improving cross‐disciplinary problem‐solving, active listening and patient participation, interprofessional communication and teamwork are fostered. Ethics serves as a framework ensuring students prioritise patient safety and comprehend professional norms where its absence could lead to conflicts jeopardising patient care.

##### 3.2.2.2 Subtheme: Student Involvement

Both undergraduate and graduate programmes inculcated IPE into their curricula. In some cases, IPE was present in early years and in others, throughout undergraduate and graduate years. Experts stated that IPE programmes were compulsory, although D1 stated that theirs carried no academic credits yet participation was high, driven by the engaging student‐centred activities, motivation and interest. Team accountability, shared experiences, community projects and exposure to other professions were factors that enhanced learning and enthusiasm. Students applied their expertise to activities such as standardised patient work, simulations, case discussions and collaborative projects both in person and online. These interprofessional engagements fostered communication, critical thinking and teamwork abilities.

Team accountability, shared experiences, community projects and exposure to other professions were factors that enhanced learning

##### 3.2.2.3 Subtheme: Facilitator/Faculty Involvement

Facilitator involvement was mostly voluntary. Facilitators with specific expertise were invited to participate and training occurred through orientations and workshops. Training focused on managing interprofessional teams, negotiating learning outcomes, overseeing the tenets of IPE, and certifying the development of expertise, creating learning materials, training standardised patients and supervising students. Facilitators engaged in weekly, bi‐weekly or monthly discussions about planning, evaluation, implementation and monitoring and were supported through inputs from various disciplines.

#### 3.2.3 Theme 3: Challenges and Opportunities

Challenges and opportunities influenced the development and implementation of IPE programmes. Many Southern African experts relayed opportunities and challenges, corroborated by international experts, related to the IPE culture at Southern African universities. However, once challenges were overcome, numerous opportunities drove, maintained and cultivated IPE.

##### 3.2.3.1 Subtheme: Challenges

Siloed working culture and professional rivalries among health sciences faculties impeded IPE programme development and implementation. Resource and logistic challenges included insufficient funding, difficulties transitioning online, lack of space, staff and knowledge limitations, training standardised patients, activity planning, scheduling, managing large groups and arranging transportation. Primarily, challenges with IPE were buy‐in, accreditation, implementation, lack of interest and understanding and justifying the relocation of credits. Implementation necessitated curriculum rework, while influencing individuals to broaden their horizons proved difficult. Challenges related to student evaluations arose with testing practical knowledge sustainability and transferability.

Challenges related to student evaluations arose with testing practical knowledge sustainability and transferability

##### 3.2.3.2 Subtheme: IPE Opportunities

A blank canvas of opportunities was created which included community engagement and led to a reduction of duplicated efforts. Due to the COVID‐19 pandemic, innovation in terms of IPE delivery improved, allowing for health schools' collaboration and sharing of learning and resources. Additional opportunities were built on capacity and skill building, exchanging ideas, research and publishing. Collaborations offered the opportunity to enhance the securing of grants or funding for research‐related ventures.

Additional opportunities were built on capacity and skill building, exchanging ideas, research and publishing

#### 3.2.4 Theme 4: Evaluation and Quality Improvements in IPE Programmes

This theme addresses assessments and measures to facilitate progress with IPE programmes, including suggestions for improvements, evaluations and dissemination of findings.

##### 3.2.4.1 Subtheme: Suggestions for Improvements

Experts concurred that IPE programmes improved through interviewing experts, which enhanced rigour and shared valuable experiences. Experts encouraged patient involvement when making decisions for optimal healthcare services. Sharing data collected from patients and communities to inform them on progress and ongoing needs was noted. IPE programmes should be designed to stimulate student engagement by fostering curiosity and relevance, giving students ownership of learning and active roles in learning experiences.

##### 3.2.4.2 Subtheme: Evaluation

Depending on the activity, experts mentioned that different types of assessments were used, including self‐developed questionnaires based on the IPEC competencies, premeasures and postmeasures, group discussions, reflective reports, peer and facilitator evaluations, online surveys and competency tools. For evaluation dissemination, encouraging faculty to publish beyond journals was emphasised. This could take the form of books, book chapters and other platforms advocating IPE, thus motivating faculty to utilise the evaluation data for improvements.

##### 3.2.4.3 Subtheme: Dissemination of Findings

Disseminating IPE findings was considered critical for scientific research and distributing innovative data in an emerging IPE field. Experts disseminated their findings through publications, conferences, workshops, poster presentations and keynote addresses. The COVID‐19 pandemic prompted a shift towards virtual conference dissemination.

Disseminating IPE findings was considered critical for scientific research and distributing innovative data in an emerging IPE field.

## 4 Discussion

We explored the perspectives of 15 international experts on IPE programme development and implementation in HPE, addressing both commonalities and context‐specificities affecting IPE in Southern Africa. We examined experts' viewpoints on IPE structures, stakeholder involvement, faculty and student engagement, challenges and opportunities and quality improvement methods.

Our findings suggest that stakeholder involvement, including institutional leadership support, was critical for effective IPE integration. Backing from regulatory bodies, deans and programme directors was highlighted as necessary to fulfil the HPE standards set out by the WHO. Most experts used existing IPE definitions while structuring their programmes around common IPEC competencies. Nevertheless, we found that there were differences in the IPE definitions, delivery and assessments. While some international experts, particularly after COVID‐19, discussed virtual delivery and classroom‐based modes, Southern African experts addressed community‐based modes of learning through rural placements and home visits. These modes were more context‐driven than general, signifying distinctions in IPE pedagogies. The syllabus and delivery modes were frequently enhanced with input from the community, facilitators and students.

These findings are consistent with previous studies that highlight the importance of teamwork, management and context‐specific IPE [1, 20]. Former studies showed that institutional buy‐in, particularly from leadership, allows for IPE to be integrated into the curricula [21]. Furthermore, the literature addressed challenges such as siloed learning, logistical constraints and inadequate faculty expertise [17, 22] which were consistent with our findings. When such challenges are overcome, substantial opportunities arise, especially for innovation, engagements and collaborations in terms of research, publishing and funding. Digital storytelling and collaborative outreach in underserved areas were community‐based IPE modes, as described by Southern African experts, reflecting culturally relevant and socially accountable pedagogies. These modes support the demands for more equity‐oriented, locally responsive IPE methods used in LMICs, as supported by the literature [5]. For LMICs, IPC skills are fostered through community‐driven IPE, even though HIC literature focused on simulation and classroom‐based modes. Thus, community‐based IPE may offer direct engagements in addressing the needs of the health system. Experts recommended strategies to improve sustainability, including launching IPE with pilot projects, programme reviews and leveraging community partnerships. Both independently created and standardised evaluation tools in line with IPC skills were discussed. Sharing programme findings through conferences, publications and journals was essential to IPE development. These results confirm previous research that promotes continuous assessment and adaptive distribution methods [23, 24].

## 5 Limitations

Although we sought perspectives of experts from different countries, our findings cannot be generalised. The study design and the sampling method may have limited the number of experts that were interviewed. However, data saturation was reached; thus, no new insights were possible. A global study including all IPE experts could provide broader perspectives on institutional viewpoints and expand identified themes. Experts who declined participation due to commitments could have contributed valuable insights, although their absence did not influence nor compromise data saturation.

## 6 Conclusion

While rising in relevance and although supported by accreditation bodies, IPE integration is context‐dependent. We provided various perspectives of IPE integration highlighting community and classroom methods together with the need for shifting online, as part of a pandemic‐driven strategy. Despite operational barriers, IPE integration through leadership, stakeholder involvement and pilot testing, can facilitate impact. Commencing with a pilot programme which later transitions to community‐oriented, leads to improvements in healthcare outcomes and capacity building. Furthermore, evaluation tools such as reports and surveys assist with improvement in programme quality and institutional support.

Our findings challenge the dominance of HICs classroom‐centric IPE programmes by emphasising practice‐oriented and culturally grounded strategies such as digital storytelling, home visits and local community collaborations of LMICs. While resource constraints are frequently cited as challenges in global literature, we found that hierarchies and rigid curricula presented greater challenges. Although experts highlighted opportunities for increased funding and positive reverberations on the community, integrating IPE in LMICs and underprivileged areas is indeed a transformative strategy for innovation and healthcare outcomes.

## Author Contributions

F.D. conceived and designed the study as a PhD student under the supervision of C.D.C. and Y.H. F.D., C.D.C. and Y.H. analysed the data. F.D. drafted the manuscript under the guidance of C.D.C. and Y.H. C.D.C. and Y.H. critically reviewed the manuscript, made significant inputs, read and agreed to the final version. All authors have contributed significantly and agree with the content of the manuscript. The requirement of authorship as specified by the journal's author guide was met, and the manuscript represents honest work.

## Ethics Statement

Ethical approval was granted by the North‐West University Human Research Ethics committee (NWU‐00430‐20‐A1).

## Consent

The authors received informed consent from participants.

## Conflicts of Interest

The authors declare no conflicts of interest.

## AI Disclaimer

We hereby declare that our submission was not AI‐generated. However, AI tools were used for summarising content, structuring, language editing and refining purposes. AI‐generated suggestions were applied responsibly and ethically.

## Supporting information


**Data S1:** Supporting Information

**Data S2:** Supporting Information

**Data S3:** Supporting Information

## Data Availability

The authors have nothing to report.
