# Supplementary material for: Shifting Interprofessional Education Pedagogies: Lessons and Implications for Africa
Source: Clin Teach. 2025 Aug 25;22(5):e70189. doi: 10.1111/tct.70189 (PMC12378144; doi:10.1111/tct.70189)
Supplement: Supplementary file 1 — Data S1: Supporting Information [file TCT-22-e70189-s003.docx]

**Addendum A – Quotes from experts during the semi-structured interview**

Quotes from experts during the semi-structured interview are presented in this addendum and are categorised according to the themes and sub-themes in the manuscript.

# Theme 1: IPE structure in Higher Education Institutions

## Sub-theme: Definition of IPE

"So, the definition that we use is based on the World Health Organization's definition, which is in 2010…which speaks to the fact that the IPE…allows two or more health professions to learn with, from and about each other, to improve collaboration and patient health outcomes.” [D6]

"We use the IPEC competencies that’s the tool that binds us together as we, we have those four big domains and all those sub-competencies as a reference point that we can use at the micro, meso and macro levels of creating different types of IPE experiences for our students” [D1]

“So, it's defined by the competencies that IPEC has set forth and the most common definition is learning from with and about each other with interprofessional students.” [D10]

"We acknowledge the CAIPE definition over into a more profession zone from, within and about one another to develop patient care" [D12]

“… it's a relatively new program at this university, I've been involved in. So, I think the term that is trying to be used rather than that the intra-professional education with regards to when we're training within the dental profession, and then inter, when we're kind of training with other healthcare professions. So, we're kind of leaning towards that way.” [D13]

“… it's really defined at this stage, in terms of community engagement, less so then in terms of treatment and assessment. In terms of client management, less, that is happening on the ground in practical modules, while technically these IHL these Integrated Health Leadership modules are theoretical modules with a community engagement component where the students do, in multidisciplinary teams and in scaling it up.” [D4]

## Sub-theme: Motivation for IPE

“… within Primary Health Care, it calls for a collaborative approach or response to health care, and so our institution took this quite seriously and decided to start an IPE unit to develop IPE and to look at possibilities within our faculty” [D3]

“… schools, in fact, have accreditation standards, which require them to have interprofessional education as part of their training” [D10]

"We have accreditation requirements…that demand that we demonstrate how we've taught interprofessional skills and provided interprofessional opportunities” [D12]

“So, the interprofessional education requirement comes from each of the governing bodies of those professions.” [D15]

“… so, the interprofessional education requirement comes from each of the governing bodies of the, of those professions. So, there's a national so for social work, we have the council for social work, education, and interprofessional education is not a requirement for the degree. Whereas in the other health sciences that were participating, there are accrediting bodies that actually require that as part of their training and degree matriculation.” [D15]

## Sub-theme: Objectives of the IPE programme

“To create a good…experience for the students…and we want to use the core competencies that are derived from the IPEC…competencies…in order to give them that best experience” [D14]

“So I think our ultimate outcome is that all our activities and programs should lead to collaborative practice.” [D3]

“… teams and teamwork, where we have a number of activities that, you know, collectively built around relationship-building values and principles of team dynamics" [D7]

“When it comes to treating patients or clients, community members and the projects that they are involved in, in communities and to be able to handle conflict” [D3]

## Sub-theme: Stakeholder Involvement

“… the main stakeholders are the deans of the college's, each College's respective Dean and, of course, students, also the faculty” [D10]

“Those are assigned by deans of their colleges, some other faculty are sometimes voluntary, for specific events or to help us develop programming on a voluntary basis” [D10]

“… we have…IPE champions in each department…so part of my role is to engage with all departments, you know, faculty and even outside of our faculty, and to identify IPE champions and, and those are the people that I then liaise with…almost on a weekly basis…so every year, I had different IPE champions in each department” [D3]

“… every year, we have a national consultation with most of the important stakeholders involved, and that would also involve the regulatory authorities…also involves local administration, because most of the projects that are carried out are under the local administration” [D7]

## Sub-theme: IPE Components

"… we would have a case study around nutrition, which is…from the diets…you would have a case study around disability, occupational therapy and physiotherapy would come…with that chronic diseases, nursing department would be the lead on that." [D6]

“a traditional World Cafe method, we use simulated patients, ethics is our, I know we've got an ethics World Cafe, and then we've got the Amazing Race” [D6]

“… the first day, we expose them to disaster management as a method of working in a team and then from that, we do role clarification exercises…the students have to triage the patients into different settings and then from there our roleplay case studies is taken" [D11]

"… they would now have to go and again identify community for a health promotion talk" [D4]

“…we have embedded the process of creating digital stories in collaboration with the community…” [D2]

“before the pandemic, we were going out, and doing home visits with older adults and having didactics and a like a debriefing kind of session” [D15]

"We do have some introductory asynchronous online learning materials built into a leadership and management course" [D1]

“ ranging from videos to podcasts to the lecturer teaching them…or via Zoom so there’s different range of things, PowerPoints” [D6]

“… we've integrated our IPE across all year levels for our students.” [D3]

“So, there's three tiers. There's novice, intermediate, and entry to practice. So, our courses are everything from two-year postgraduate, nutrition and dietetic course to a five-year medicine course. So, we don't say everyone does novice in year one and does entry to practice in year four. So, it's intentionally, each profession deems where students are at from novice, intermediate, entry to practice.” [D12]

# Theme 2: Faculty and Student Involvement

## Sub-theme: Coordination

“… more teamwork and more team care and also that patient-centred approach where the whole team is able to provide care for that patient rather than it being in a segregated way, better understanding of utilising team roles” [D13]

“… provide and promote a team approach to patient care and health care management” [D10]

“… the real focus is on IPC interprofessional collaboration, for community engagement, in terms of IPE, they learn together and so there is some it's professional learning, and sharing ideas in class and they in this team together, they have to know problem solve and have their rules of engagement and how they're going to go about things in dividing up their roles and writing the projects and all those submissions, and their evidence of what they've done. All has to be done in a team and that leads to a lot of the interprofessional education in the role sharing…” [D4]

## Sub-theme: Student Involvement

"We have two courses, each of which is a semester-long and all students are required to be in that " [D1]

“I think one of the reasons that they are motivated is maybe the time commitment for a lot of these IPE activities is not burdensome. It's a few hours of prep work, and a few hours of collaboration with other people and they find it a joy” [D1]

“They will carry out some online activities initially alone, and then as part of a group and produce either posters or presentations or group work, which they then post on to some of the virtual learning environments” [D8]

“You do have to put in sessions on team working, and the different scopes and what the different team members do” [D13]

“They are there to be well you know, an MD, a nurse, a public health professional in training…it's a chance to take everything that they've learned, that they’ve learnt everything they know, everything they're able to do so far in that discipline, and put it to work” [D1]

## Sub-theme: Facilitator/Faculty involvement

“So we basically had task team meetings. So we had, for example, monthly meetings where we actually got together and we developed the programme before then” [D6]

“… it's still on a voluntary basis, and more or less trying to purposively target faculty with specific skills or specific expertise that can be brought into the system” [D2]

#

# Theme 3: Challenges and Opportunities

## Sub-theme: Challenges

“…. professional rivalries, well known so, there was quite a bit to overcome those hardcore professional identities” [D7]

"The participation of especially the faculty members is on a very voluntary basis, and that sometimes becomes very frustrating because the people in the department don't necessarily realise how time-intensive it is to plan this, and how much effort the staff members put in" [D11]

“Funding is another challenge, because the faculty, you have to design something where there's no budget for” [D3]

“So basically, two big, big, big challenges is the current existing culture, surrounding IPE and thinking about IPE, and then to change that. And secondly, is to get people on board so that they buy into the necessity and importance of IPE.” [D5]

" …. how sustainable is that knowledge? And how transferable is that knowledge to a practical context? " [D2]

## Sub-theme: IPE Opportunities

"Now that more staff across the different courses linked in really, we know each other, we share work, and that means less duplication" [D12]

“… I will note a trend towards virtual IPE since COVID-19, as well” [D1]

“….. networking, in the global spaces, there’s a lot of other universities globally, that's involved with IPE now” [D6]

" I've now seen many grant calls; they want to see how people work across institutions. They also wanted to see how people work collaboratively, so they won't accept the funding proposal if you can't show how you are working across departments, across faculties and across institutions." [D3]

# Theme 4: Evaluation and quality improvements in IPE programmes

## Sub-theme: Suggestions for improvements

"… a strong representation from each health profession has to be around the deciding table, and patients have to be around the deciding table, and some student reps have to be there." [D12]

"We always negate the process of going back to where we collected the data, where we did the interventions and bring people in a town hall and actually share this is what has happened, this is what we're doing, and this is what we still need and these are the successes" [D2]

“… a student must have the ownership of learning, and ownership of learning happens if they find that it is relevant to their profession” [D7]

## Sub-theme: Evaluation

“We also rely a lot on student reflective writing to have them address the questions that we want them thinking about” [D1]

“… don't have the time as faculty to go back over that data and utilise it to continuously improve our programmes and probably the only time we do that is when we're motivated to do some sort of publication on the activities that we've been engaging in together" [D1]

“So, it’s basically publication, conference presentation and we also consider doing a book chapter” [D5]

## Sub-theme: Dissemination of findings

"… we've published in Medical Teacher, I think, we've published in the Journal of Interprofessional Care. We've published extensively in the African Journal of HPE" [D6]

"We are presenting a workshop for colleagues…I also have a presentation at SAAHE on our findings in this research; this is also presented at the ISOTL conference and AIMEE as well" [D5]

## Sub-theme: Dissemination of findings

“… CV has popped up quite a bit…during COVID…because of all the virtual conferences, but we've been disseminating all over the place at conferences” [D1]

“… we would often invite those stakeholders to attend the students’ presentations… it's critical for stakeholders to be involved with what the students are doing and to give their feedback, feedback and input throughout the time that the students are there.” [D3]
