# Supplementary material for: Shifting Interprofessional Education Pedagogies: Lessons and Implications for Africa
Source: Clin Teach. 2025 Aug 25;22(5):e70189. doi: 10.1111/tct.70189 (PMC12378144; doi:10.1111/tct.70189)
Supplement: Supplementary file 2 — Data S2: Supporting Information [file TCT-22-e70189-s001.docx]

## Addendum B: Semi-structured interview schedule for individual interviews

**1. Research Title:**

Developing an interprofessional education programme for a health science faculty.

**2. Qualitative Interview Introduction**

The length of the session is 60-70 minutes. The aim of this session will be to explore criteria associated with effective IPE training programmes and the challenges encountered with implementing IPE programmes.

**3. Protocol of the researcher**

The researcher will:

1. Introduce herself.
2. Seek consent to record (for purpose of record keeping and report for ethics committee).
3. Thank the participant for his/her inclination and time to participate in this study.
4. Make sure that the informant is comfortable and ready for the individual interview.
5. Describe the aims and objectives of the research.
6. Notify the informant of his/her role in this phase of the research.
7. Explain the need of the interview and the time required to gather the information.
8. Verify the ethical considerations for the semi-structured interview. Confirm the use of the audio recorder and the purpose of using the audio recorder whilst maintaining privacy and confidentiality.
9. Confirm voluntary participation and withdrawal at any time without facing negative consequences.
10. Request the participant for any comments, queries or concerns and provide clarification thereof.

**4. Verbal Consent and Re-consent**

Indicate to the informant of their written consent and request verbal consent if they agree to participate in this study. Should the participant not confirm, they may withdraw from the research without any consequences. Obtain re-consent regarding the recording of the interview conducted on the online platform.

**5. Commencing the semi-structured interview schedule**

Different communication techniques such as probing or further questioning for clarification, will be used for the possibility of gaining a better insight into the criteria associated with effective IPE training programmes and challenges encountered with implementing IPE programmes.

**6. Interview Schedule**

**Schedule based on the framework of El-Awaisi (2016).**

*(Addendum C may be used to follow-up during the interview if the individuals are not providing all the information).*

**Question 1:** Please tell me how you started with IPE at your institution, and what was the objective of bringing IPE into your institution.

**Question 2:** Please share with me how IPE is implemented at your institution. Please be as specific as possible, I may ask follow-up questions should I need clarification or more information.

**Question 3:** Please explain to me what role the students plays in your IPE programme (roles, activities, assessment, experiences, feedback, etc)

**Question 4:** Please explain to me what role the lecturers play in your IPE programme.

**Question 5:** Please explain to me what other stakeholders are involved with your IPE programme, what are their roles, and how do you collaborate with them?
