# Supplementary material for: Shifting Interprofessional Education Pedagogies: Lessons and Implications for Africa
Source: Clin Teach. 2025 Aug 25;22(5):e70189. doi: 10.1111/tct.70189 (PMC12378144; doi:10.1111/tct.70189)
Supplement: Supplementary file 3 — Data S3: Supporting Information [file TCT-22-e70189-s002.docx]

## Addendum C: Checklist for individual interviews based on the El-Awaisi et al (2016) framework.

| **Key Areas** | **Yes** | **No** |
| --- | --- | --- |
| How the IPE programme started. |  |  |
| Who the main stakeholders were then and now? |  |  |
| How IPE is defined in their programme. |  |  |
| What the values and standards are of IPE in their programme. |  |  |
| What outcomes are stated for thier IPE programme. |  |  |
| How the participation and selection of students and faculty was done. |  |  |
| Do they implement IPE according to specific themes? If so, how did they decide on those themes?  (a) Who took part in designing the cases and activities for IPE?  (b) How did they collaborate?  (c) Challenges and opportunities? |  |  |
| What their programme looks like in terms of level and stages and how the levels and stages were determined. |  |  |
| What kind of learning activities are the students involved in and how learning was facilitated? |  |  |
| How students were kept involved and motivated and how the expectations and experiences of students were raised. |  |  |
| How student feedback was assessed and utilised. |  |  |
| How the impact of the IPE on the community (if any) was evaluated. |  |  |
| How experiences of the health students were shared (with who). |  |  |
| Any other comments to share?  Comment about study and what do they think? |  |  |
